# Drought Tolerance in Wild Plant Populations: The Case of Common Beans (*Phaseolus vulgaris* L.)

**DOI:** 10.1371/journal.pone.0062898

**Published:** 2013-05-03

**Authors:** Andrés J. Cortés, Fredy A. Monserrate, Julián Ramírez-Villegas, Santiago Madriñán, Matthew W. Blair

**Affiliations:** 1 Evolutionary Biology Center, Uppsala University, Uppsala, Sweden; 2 Laboratorio de Botánica y Sistemática, Universidad de los Andes, Bogotá, Colombia; 3 Centro Agropecuario, Servicio de Enseñanza Nacional, Buga, Colombia; 4 School of Earth and Environment, University of Leeds, Leeds, United Kingdom; 5 CGIAR Program on Climate Change, Agriculture and Food Security (CCAFS), Cali, Colombia; 6 Department of Plant Breeding, Cornell University, Ithaca, NY, United States; New York State Museum, United States of America

## Abstract

Reliable estimations of drought tolerance in wild plant populations have proved to be challenging and more accessible alternatives are desirable. With that in mind, an ecological diversity study was conducted based on the geographical origin of 104 wild common bean accessions to estimate drought tolerance in their natural habitats. Our wild population sample covered a range of mesic to very dry habitats from Mexico to Argentina. Two potential evapotranspiration models that considered the effects of temperature and radiation were coupled with the precipitation regimes of the last fifty years for each collection site based on geographical information system analysis. We found that wild accessions were distributed among different precipitation regimes following a latitudinal gradient and that habitat ecological diversity of the collection sites was associated with natural sub-populations. We also detected a broader geographic distribution of wild beans across ecologies compared to cultivated common beans in a reference collection of 297 cultivars. Habitat drought stress index based on the Thornthwaite potential evapotranspiration model was equivalent to the Hamon estimator. Both ecological drought stress indexes would be useful together with population structure for the genealogical analysis of gene families in common bean, for genome-wide genetic-environmental associations, and for postulating the evolutionary history and diversification processes that have occurred for the species. Finally, we propose that wild common bean should be taken into account to exploit variation for drought tolerance in cultivated common bean which is generally considered susceptible as a crop to drought stress.

## Introduction

Common bean (*Phaseolus vulgaris* L.) is a key source of nutrients and dietary protein for over half a billion people in Latin America and Africa and nearly 4 million hectares are grown in zones where drought is severe, such as in northeastern Brazil, coastal Peru, the northern highlands of México and in dry parts of Africa [1]. Therefore, increasing drought tolerance in common bean commercial varieties is highly desirable. A considerable reservoir for this task may be available in the wild and cultivated collections of common bean, as can be suggested by their high genetic diversity and phenotypic variability [2], [3], [4].

Wild common bean is an annual, viney plant that germinates among small trees and shrubs in forest clearings or in disturbed environments with the onset of seasonal rains [5], [6]. Specifically, the growth cycle of the wild common bean is from 8 to 10 months in length. Hence, in tropical bimodal rainfall regions wild common bean is subjected to a mid-term drought, while in sub-tropical unimodal rainfall regions wild beans can be subjected to more prolonged periods of water stress. These drought stresses are characteristic of environments in the inter-Andean valleys of the Andes in South America and in northern parts of Mesoamerica especially the volcanic axis and mountains of Mexico [7], [8]. Although wild common bean is promising in terms of drought tolerance, the evaluation of drought physiology traits in wild populations would be impractical due to long growth cycle and seed dehiscence [7]. Consequently, alternative methods should be explored in order to discover potential drought tolerance sources in wild populations based on the characteristics of their natural habitats as done for other species [4], [9], [10], [11].

In this sense, potential evapotranspiration (PET) modeling is a powerful tool to predict drought severity for a geographic site or the accessions’ origin, so as to identify sources of drought tolerance in cases in which no phenotypic evaluations are available [12]. PET is a theoretical value that aims to characterize the quantity of water that will flux from the soil-biosphere system toward the atmosphere given the effects of evaporation and transpiration and provided that soil water is enough to supply the demand[13]. PET can be calculated purely with climatic variables provided that the hypothetical effect of each of the variables for evaporation and transpiration is known [14], [15].

Calculations of PET consider that transpiration and evaporation are proportional to temperature. Two lines of evidence support this assumption. First, increasing temperatures leads to an increase in the maximum density of water vapor (until the air is fully saturated with water vapor), and coincidentally to a relative decrease in humidity (relative humidity = real density of water vapor/maximum density of water vapor). Relative air humidity is proportional to the water potential gradient between the plant-soil system and the surrounding air. Hence, there would be a net flux of water from the plant toward the surrounding air during drought stress events. In addition, temperature is proportional to the energy transferred and is a necessary variable for understanding the phase change between liquid and gaseous water [15], [16]. PET modeling also considers that evaporation and transpiration are proportional to radiation because radiation is proportional to the energy transferred from sunlight to plants. Radiation is thus a necessary variable for understanding phase change. Finally leaf conductance is proportional to radiation, at least for C3 plants due to the modulation of stomata opening [17]. The two common non-intensive methods to calculate PET based on temperature and radiation are the Thornthwaite method [16] and the Hamon method [15]. The former considers the effects of both temperature and radiation explicitly, while the latter is purely based on temperature effects.

To determine net water flux given the effects of temperature and radiation, habitat drought index can be calculated comparing the values for PET and for precipitation (P). In particular, three scenarios can be recognized: PET and P are equivalent, PET is higher than P, and P is higher than PET [18], [19]. Two considerations must be taken into account before deciding on the biological meaning of each scenario. First, PET and P estimations in a specific period of time are based on stochastic variables. Second, the period of time considered for the previous calculations and analysis will determine if the plant is actually subjected to significant water stress or not. For instance, PET higher than P implies that drought stress is a constant if PET and P are measured in time scales in which a water deficit impacts the plant’s physiology. In contrast, P higher than PET presumes that the plant does not experience water stress during the time scale of measurements. However, it is notable and counter-intuitive that PET equals P does not predict an absolute stress condition because of the stochastic components of both PET and P, and because of the soil water holding capacity [20], [21].

The objectives of this research were 1) to evaluate the environmental variability in collection site habitats for a core collection of wild common bean that had been previously fingerprinted for genepool and sub-population structure and 2) to determine through two (Thornthwaite vs. Hamon) methods of PET modeling the extent of drought tolerance, the correlation of drought tolerance with collection site characteristics and the association of drought tolerance estimates based on environmental data for the collection site with genetic population and sub-population structure of the wild bean collection. We also evaluated whether the classification of geographical distribution based on drought stress was dissimilar between wild and cultivated common beans, and if their patterns of geographical variation could be determined by local adaptation to hydrological regimes or by evolutionary inertia.

## Materials and Methods

### Plant Material

A total of 104 wild common bean accessions were considered in this study. All the genotypes were loaned by the Genetic Resources Unit at the International Center for Tropical Agriculture and are preserved under the treaty for genetic resources from the Food and Agriculture Organization, hereafter abbreviated as the FAO collection. In addition, information on drought tolerance of 297 cultivars of common beans from Pérez *et al*. [22] were considered to compare distribution of cultivated and wild common beans. These two reference collections were selected to be a representative sample of genepools and races, based on a subset of core collections for wild [23] and cultivated [24] beans. Their analysis with neutral molecular markers has also been previously described [25]. Finally, the definition of wild genetic sub-populations is according to Blair *et al*. [26] and Broughton *et al.* [27]. Geographic information was provided for each accession by the Genetic Resource Unit (http://isa.ciat.cgiar.org/urg/main.do). In order to estimate drought tolerance for wild common bean, 19 bioclimatic variables were downloaded from WorldClim (http://www.worldclim.org) and they were recorded for each wild accession point (table 1).

**Table 1:** Contribution (%) of each bioclimatic variable to the PCA analysis.

Code	MainVariable	Bioclimatic Variable	Total Variables (T)			Only Precipitation Variables (P)			Drought-related variables (*, S)		
			F1+	F2	F3	F1	F2	F3	F1	F2	F3
			40.41%	30.17%	13.04%	57.16%	26.27%	9.28%	42.02%	34.28%	11.88%
bio_1	Temperature	P1. Annual Mean Temperature*	4.08	**11.26**	1.19				4.52	**22.73**	0.94
bio_2		P2. Mean Diurnal Temp. Range (Mean(period max-min))	7.87	1.11	0.01						
bio_3		P3. Isothermality (P2/P7)	4.58	3.23	**11.63**						
bio_4		P4. Temperature Seasonality (Coefficient of Variation)*	6.47	1.99	7.25				6.72	4.06	**34.78**
bio_5		P5. Max Temperature of Warmest Period*	0.01	**16.38**	0.00				**15.80**	7.48	1.45
bio_6		P6. Min Temperature of Coldest Period	***10.17***	2.03	3.23						
bio_7		P7. Temperature Annual Range (P5–P6)	**9.07**	2.59	2.77						
bio_8		P8. Mean Temperature of Wettest Quarter	0.85	***14.25***	0.02						
bio_9		P9. Mean Temperature of Driest Quarter*	6.01	7.67	2.16				1.81	**24.28**	5.31
bio_10		P10. Mean Temperature of Warmest Quarter*	0.91	**15.03**	0.00				9.95	**15.02**	1.75
bio_11		P11. Mean Temperature of Coldest Quarter	7.68	4.83	4.42						
bio_12	Precipitation	P12. Annual Precipitation*	**8.58**	0.06	**10.41**	**18.14**	5.53	2.13	5.96	**12.26**	4.14
bio_13		P13. Precipitation of Wettest Period	5.29	0.83	**16.18**	11.32	***21.87***	1.59			
bio_14		P14. Precipitation of Driest Period*	6.10	3.75	2.36	**16.73**	7.67	2.59	**15.97**	5.59	2.29
bio_15		P15. Precipitation Seasonality (Coefficient of Variation)*	3.51	7.94	2.13	6.97	**24.60**	0.01	**16.48**	0.62	9.38
bio_16		P16. Precipitation of Wettest Quarter	5.11	0.85	**16.20**	11.52	***20.38***	3.91			
bio_17		P17. Precipitation of Driest Quarter*	6.44	3.70	1.16	**16.15**	9.92	1.65	**15.81**	6.03	1.02
bio_18		P18. Precipitation of Warmest Quarter	2.17	1.09	**18.58**	10.39	1.90	**51.51**	6.99	1.94	**38.93**
bio_19		P19. Precipitation of Coldest Quarter	5.10	1.39	0.29	8.77	8.12	**36.61**			

+ Three categories (F1–F3) are used to analyze the 19 bioclimatic variables. Three main components and the percentage of explained variance are indicated for each category.

* Variables used in the third analysis (selected because of being strictly drought-related variables).

- Bold numbers: Variables with significant contribution in the definition of the respective component and pertinent for drought stress estimation.

- Bold and italic numbers: Variables with significant contribution in the definition of the respective component but not conceptually pertinent for drought stress estimation.

### Multivariate Analysis

Visual correlation between population structure previously accessed for the wild collection and drought stress was assessed by overlaying the dot-map distribution of the accessions with the precipitation pattern using ESRI’s ArcView (ESRI, Inc.). Furthermore, the variation of precipitation and temperature in different regions along the latitudinal pattern was assessed using DIVA-GIS 7.1.6 [28]. With the aim of determining which variables were useful for estimating the drought stress of each accession habitat, the all 19 bioclimatic variables as well as two subsets of these (one of them including precipitation-related variables and the other one including drought-related variables – see table 1) were subjected to scatter plot, principal components and cluster analysis (Pearson’s correlation coefficient (r), Spearman's rank correlation coefficient (ρ) and middle joint method). The variables in the sub-set of drought-related were chosen according to their relation to mean temperature during the warmest period and precipitation during the driest period, which both are associated to drought events. For example, annual precipitation and precipitation of the driest period indicate long- and short-term stress, respectively. Graphics were revised and edited in SigmaPlot (Systat-Software, Inc.). Population structure was considered based on results from Blair *et al*. (2009) and an analysis of variance was carried out to recognize differences between populations using XLSTAT 7.5.2 (Addinsoft, Inc.) and STATISTIX 8 (Statistix: Analytical Software, Inc.).

### Potential Evapotranspiration and Drought Index Calculation

Monthly potential evapotranspiration was calculated for each accession using the bioclimatic information and the Thornthwaite [14], [16] and Hamon [15] approximations. Thornthwaite equations considered the effects of temperature and radiation on the calculation of the potential evapotranspiration (PET). In particular, we used the following equation (for the month “j”):
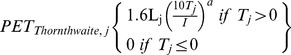



Where 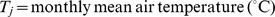


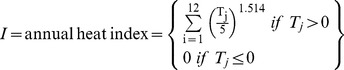


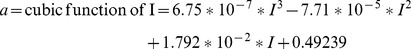


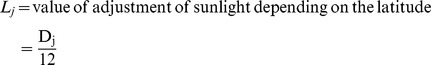


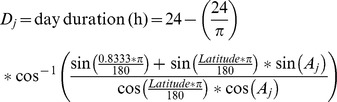


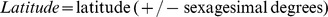


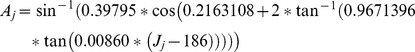


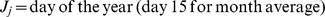



On the other hand, Hamon approximation estimated PET based exclusively on temperature effects. In this sense, we used the following equation (for the month “j”):
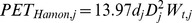



Where 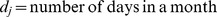


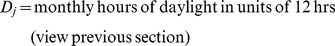


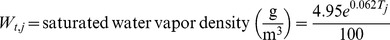


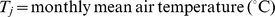



Monthly drought indices were obtained by comparison of these PET estimators with monthly total precipitation (P). The following drought index (DI) ratio was used (for the month “j” and the PET calculation approximation “i”):
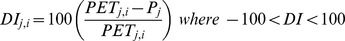



Finally, normalized annual mean drought index (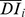) and annual maximum drought index (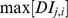) were determined for each accession following both strategies to calculate the PET. The first pretends to analyze long term theoretical habitat drought, while the second one explores short term and sporadic drought.

## Results

### Clusters of Environmental Variability

**Figure 1:**
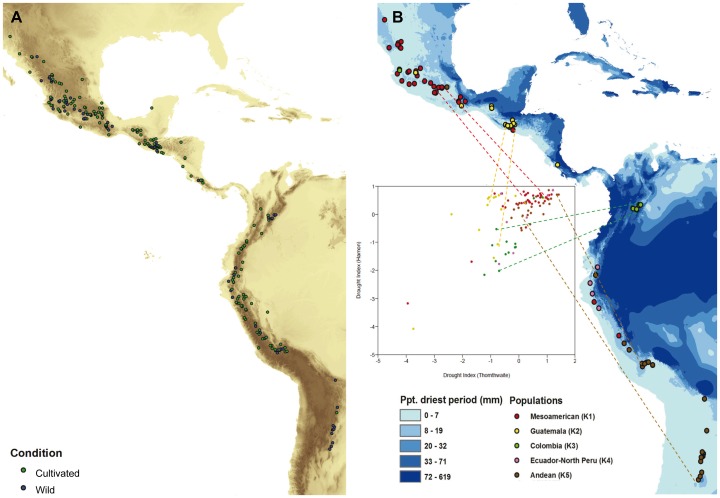
Geographic distribution of wild (104 accessions) and cultivated (297 accessions) common bean accessions (A), and precipitation during the driest period along the geographic range of wild common bean (B). A dispersion diagram between the estimated drought index using the potential evapo-transpiration (PET) of Thornthwaite and the estimated drought index using the PET of Hamon is presented in B. Populations definition as in Blair *et al.* [26] and Broughton et al. [27].

**Figure 2:**
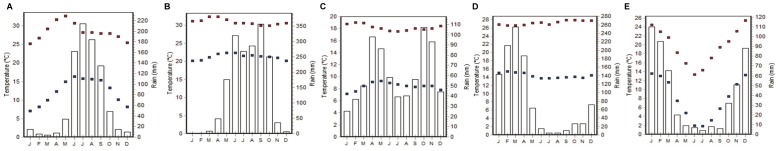
Temporal variation of precipitation (bars), maximum temperature (red squares) and minimum temperature (blue squares) in different representative regions: A. Mexico (−102° latitude, 20° longitude), B. Guatemala (−90°, 14°), C. Colombia (−74°, 4°), D. Ecuador-North Peru (−80°, −4°) and E. Argentina (−65°, −24°).

The wild accessions were distributed among different precipitation regimes that followed a latitudinal gradient from North to South America. Thus, accessions from Central America and northwest South America (regions near the equator) were associated with higher precipitation (annual precipitation and precipitation of the wettest quarter), while accessions from northern Mexico and central Andean regions found in Argentina, Bolivia and Peru (near the tropics of Cancer and Capricorn) were associated with lower precipitation (figure 1 and 2). Additionally, wild common beans occupied more geographical regions with extensive drought stress than cultivated accessions (t = 3.21, p-value = 0.0014, n = 399).

Five sub-populations have been recognized within wild common beans: Mesoamerica (Mexican wilds), Guatemala, Colombia, Ecuador and North Peru, and Andean (wilds from Argentina, Bolivia and southern Peru) sub-population *per* Blair et al. [26] and Broughton *et al*. [27]. The clustering of genetic groups within each of the wild sub-populations also followed a latitudinal gradient except for a pair of accessions collected in Peru that belonged to the Mesoamerican sub-population and two accessions collected in Mexico that belonged to the Colombian and Andean sub-populations. In these cases, the precipitation regime at the collection sites for these accessions was similar to the precipitation pattern associated with the whole sub-population (figure S1). For example, Mesoamerican and Andean wild populations were generally restricted to low precipitation habitats, whereas Colombia and Guatemala populations were distributed in higher precipitation habitats, although these could be found along wider gradients of total rainfall (table 1 and 2).

**Table 2:** Pairwise comparisons of significant variation for each bioclimatic variable, component and drought severity estimator in relation with population structure (p-value<0,001).

	P1	P2	P3	P4	P5	P6	P7	P8	P9	P10	P11	P12	P13	P14	P15	P16	P17	P18	P19	DIT	DIT max	DIH	DIH max	F1P	F2P	F1S	F2S
M (K1)	A	A	–C	A	A	–B	A	A	A	A	A	–B	–B	–B	A	AB	–B	–B	–B	AB	A	A	A	–B	A	A	A
G (K2)	A	–B	–BC	–B	–B	AB	–B	–B	A	–BC	A	A	A	–B	–B	A	–B	A	–B	–C	–BC	–B	–BC	A	A	–BC	A
C (K3)	A	–B	AB	–B	–B	A	–B	AB	A	AB	A	AB	–BC	A	–C	–BC	A	AB	A	–BC	–C	AB	–C	A	–C	–C	A
E (K4)	A	–B	A	–B	–B	AB	–B	AB	A	–BC	A	–BC	–BC	AB	AB	–BC	–B	AB	AB	AB	ABC	A	ABC	AB	–B	–BC	A
A (K5)	–B	A	–C	A	–B	–C	A	–B	–B	–C	–B	–C	–C	–B	–B	–C	–B	–B	–B	A	AB	A	AB	–B	–B	–B	–B

Variable abbreviations: P1–P19: main variables as defined in table 1, DIT: Annual Mean Drought Index (Thornthwaite), DIT, Max: Maximum Drought Index (Thornthwaite), DIH: Annual Mean Drought Index (Hamon), DIH, Max: Maximum Drought Index (Hamon), F1P, F2P: Main two factors for bioclimatic precipitation variables (P12–P19), F1S, F2S: Main two factors for drought-related variables.

Kruskal-Wallis tests were applied in all cases except for DI (Drought Index) estimations, where an ANOVA followed by a Tukey’s-b post-hoc test was used. A, B and C are different ranks. Populations with more than one letter could not be assigned to a single rank. Mean for each variable for each population: A>B>C.

Bold variables: Drought-related variables. Selected variables for further analysis based on their conceptual power to describe drought tolerance.

**Figure 3:**
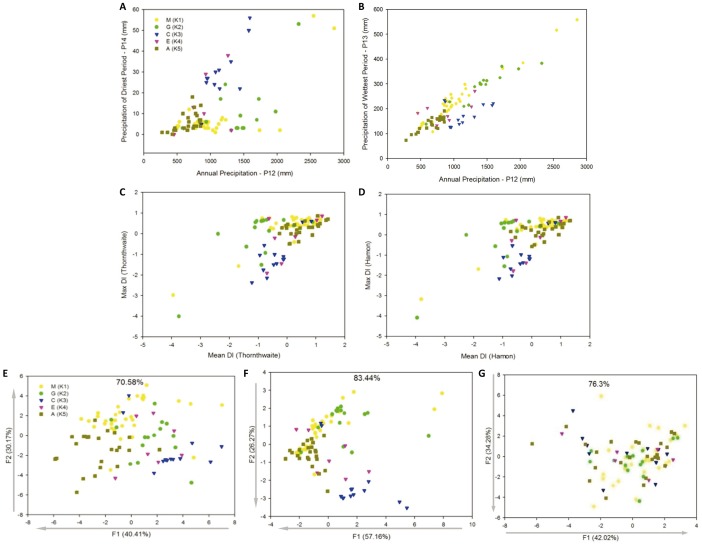
Scatter plots for: A.mean annual precipitation (P12) and precipitation of the driest period (P14), B. mean annual precipitation (P12) and precipitation of the wettest period (P13), C. mean and maximum Thornthwaite Drought Index (DI), D. mean and maximum Hamon DI, E. two main components of the PCA for all bioclimatic variables (P1–P19– table 1), F. two main components of the PCA for precipitation related bioclimatic variables (P12–P19– table 1), and G. two main components of the PCA for drought-related bioclimatic variables (table 1). Arrows indicate the increase in the estimated drought stress for each component. Wild populations: M: Mesoamerican, G: Guatemala, C: Colombia, E: Ecuador-North Peru and A: Andean. Numbers in E, F and G are percentage of explained variation by each component.

Precipitation of the driest and wettest periods, mean and maximum Thornthwaite Drought Index, and mean and maximum Hamon Drought Index of each sub-population in biplot analysis confirmed the separation of the sub-populations into ecological niches (table 2 and figure 3). Each of the main components (F1-3) showed a slightly different pattern of environmental variation in relation with population structure, as is depicted in table 2. Among the three analytical sets which were considered (all bioclimatic variables, precipitation variables and drought-related variables) the first explained population structure the best (see dashed divisions in table 2 and variables in bold). The behavior of the variables in predicting wild accessions sub-grouping was confirmed by the clustering analysis with all bioclimatic variables (figure S2). However, the resolution provided by the PCA and clustering analysis to discern between natural populations was not comparable with that achieved by the use of drought indices. Analysis of variance (table 2) confirmed these observations.

### Comparison of Methods to Estimate Drought Tolerance

Among the main components of the PCA analysis for all bioclimatic variables, the first two were not determined by variables conceptually meaningful in the context of drought stress estimation, such as mean temperature of driest quarter and precipitation of warmest quarter (table 1). However, the third component was significantly determined by these variables, although it explained only 13% of total variation. Annual precipitation was present in all the components and therefore did not offer discriminatory power. Variables related with drought stress contributed considerably to the first components of the PCA analysis for the two additional subsets of bioclimatic variables (precipitation-related variables and strictly drought related variables). The contribution of drought stress relevant variables to the other two components of both subsets was lower. We observed that the first component of the PCA analysis for precipitation variables was a good estimator of long term habitat drought stress, while the second component for the drought-related variables was a good estimator of short term and sporadic drought stress.

This pattern was corroborated by Pearson’s correlation (r) and Spearman's rank correlation coefficients (ρ) tests for the significant PCA components with annual mean Drought Index and maximum Drought Index (from Thornthwaite and Hamon), respectively (table 3). The three main components were correlated with temperature and precipitation variables, whereas drought indices based on evapotranspiration were only correlated with precipitation variables, as would be expected. Pearson’s and Spearman's coefficients provide the same pattern of correlation, which means that the assumption of normality is not a strong premise for our other analysis.

**Table 3:** Pearson’s correlation coefficients (r – above the diagonal) and Spearman's rank correlation coefficients (ρ – below the diagonal) among some representative climatic variables, components and drought severity estimators.

	DIT	DIT max	DIH	DIH max	F1T	F2T	F1P	F2P	F1S	F2S	P12	P14	P1	P9
DIT		**0.73**	**0.99**	**0.74**	**−0.71**	**0.4**	**−0.9**	−0.14	**0.73**	**−0.39**	**−0.91**	**−0.7**	0.03	−0.14
DIT max	**0.55**		**0.73**	**1**	**−0.6**	**0.59**	**−0.82**	**0.43**	**0.88**	**−0.29**	**−0.57**	**−0.96**	0.15	0.02
DIH	**0.99**	**0.56**		**0.75**	**−0.67**	**0.45**	**−0.89**	**−**0.13	**0.76**	**−0.33**	**−0.89**	**−0.7**	0.09	−0.07
DIH max	**0.57**	**1.00**	**0.58**		**−0.59**	**0.6**	**−0.83**	**0.41**	**0.89**	**−0.28**	**−0.58**	**−0.96**	0.17	0.03
F1T	**−0.71**	−0.33	**−0.67**	**−**0.33		0	**0.84**	**−**0.01	**−0.47**	**0.84**	**0.81**	**0.68**	**0.56**	**0.68**
F2T	0.41	**0.66**	0.46	**0.68**	−0.01		**−0.28**	**0.58**	**0.85**	**0.5**	−0.06	**−0.46**	**0.8**	**0.66**
F1P	**−0.89**	**−0.60**	**−0.86**	−0.60	**0.83**	−0.25		0	**−0.72**	**0.6**	**0.91**	**0.87**	0.18	**0.29**
F2P	−0.17	0.46	−0.14	0.46	0.15	**0.60**	0.13		**0.47**	**0.21**	**0.34**	**−0.4**	**0.32**	**0.25**
F1S	**0.69**	**0.82**	**0.72**	**0.83**	−0.36	**0.89**	**−0.59**	0.44		0	**−0.5**	**−0.82**	**0.44**	**0.28**
F2S	−0.38	−0.01	−0.32	−0.01	**0.83**	0.47	**0.59**	0.35	0.12		**0.65**	**0.44**	**0.88**	**0.91**
P12	**−0.89**	−0.33	**−0.86**	−0.34	**0.82**	−0.03	**0.91**	0.41	−0.37	**0.68**		**0.63**	**0.3**	**0.4**
P14	**−0.57**	**−0.94**	**−0.57**	**−0.94**	0.45	−0.50	**0.69**	−0.38	**−0.70**	0.20	0.43		0	0.12
P1	−0.01	0.34	0.05	0.34	**0.54**	**0.77**	0.22	0.39	0.49	**0.85**	0.36	−0.14		**0.95**
P9	−0.16	0.24	−0.10	0.24	**0.66**	**0.65**	0.35	0.35	0.36	**0.91**	0.48	−0.03	**0.96**	

Bold: significant values: <0.05 for r or <−0.5 and >0.5 for ρ.

DI_T_: Normalized Annual Thornthwaite Drought Index.

DI_H_: Normalized Annual Hamon Drought Index.

DI _max N_: Normalized Maximum Month Drought Index (Thornthwaite (T) or Hamon (H)).

F#i: Two main components using all bioclimatic variables (i = T), only precipitation variables (i = P), or only drought-related variables (i = S) (table 1).

Original Control Bioclimatic Variables: P12: Annual Precipitation, P14: Precipitation of Driest Period, P1: Annual Mean Temperature, P9: Mean Temperature of Driest Quarter.

Finally, in the evaluation of the two indices of potential evapotranspiration obtained from the bioclimatic variables we found both to be similarly predictive. The correspondence between Thornthwaite and Hamon drought index estimators (normalized annual mean and annual maximum) was indicated by the correlation analysis where r-values were of 0.99 and 1.00 showing them to be nearly analogous estimators. Additionally, the value of these indices to detect long and short drought stress for a habitat based on potential evapotranspiration is shown by the correlation with mean and maximum annual precipitation (Table 1 and figure S2).

## Discussion

The evaluation of drought physiology traits in wild common bean populations would have been impractical due to their long growth cycle and low biomass. Hence, the ecological analysis of wild bean accessions geographical origin performed here was useful in successfully predicting the drought tolerance of these genotypes in a case where other sources of information were not available. Two particular issues were considered: the way in which ecological variation is structured into natural populations, and the ideal and unequivocal estimator of drought tolerance.

### Ecological Diversity is Structured along the Populations of Wild Bean

The analysis disclosed three non-overlapping categories of drought tolerance associated with population structure and extensively correlated with a latitudinal pattern. Correlation between ecology and geographical distance is a common phenomenon in natural populations which responds to isolation of sub-populations [29]. This is a consequence of independent evolution in different subpopulations of a species evolving towards adaptation to specific microclimatic conditions [30], [31]. Moreover, random accumulation of genetic variability is uneven along populations because of genetic drift and bottlenecks. Consequently, the genetic resources available in each population to deal with new or old selective forces are dissimilar between groups or demes. The evolution pathway followed by each population is therefore unique [32]. This is the foundation of the adaptive radiation hypothesis according to which meta-population structure will favor adaptive and diversifying selection.

Previous research from Tiranti and Negri [33] demonstrated that selective microenvironmental effects play a role in shaping genetic diversity and structure in common bean wild accessions. In this study we have confirmed that ecological diversity is associated with structuring into natural populations in wild beans. In contrast, cultivated accessions of common bean are mainly structured into races, with less explanation of diversity by latitudinal shifts or gradients. This difference between wild and cultivated common bean might be a consequence of continental level rainfall patterns. Specifically, in tropical environments near the equator with bimodal rainfall a mid-season dry period occurs that can last two to four weeks. In contrast in the sub-tropics, a dry period of three or more months can occur. In response to this mid-cycle drought of the sub-tropics, wild *P. vulgaris* enters a survival mode of slow growth and reduced physiological activity until rainfall resumes and flowering occurs [7]. Cultivated beans on the other hand are less frequently subjected to these environmental pressures and tend to mature in a shorter length of time.

Interestingly, we observed that wild common bean occupy more geographical regions with extensive drought stress than cultivated accessions. Those regions include the arid areas of Peru, Bolivia and Argentina, and the valleys of northwest Mexico. In addition, it is necessary to emphasize that the correlation between population structure and climatic variability could also be a partial consequence of other correlated latitudinal variation not necessarily driven by day length and temperature. Hence, population structure as well as climatic variability constraints must be taken into account to analyze genetic variation in relation with theoretical drought stress of each habitat.

In summary, we have detected a broad habitat distribution for wild common beans that is useful for drought tolerance. Cultivated common bean is traditionally considered susceptible to drought, but that seems not to be the case for wild common beans. In addition, some differences must exist between the adaptations of wild populations to arid regimes which are reflected in the sub-populations found in different ecologies. Several of them are valuable for plant breeding. Therefore, we propose, as was suggested by Acosta *et al*. [34], that wild common bean be taken into account to exploit variation for drought tolerance, however care is needed to avoid the reduction in yield associated with the wild bean genotype.

### Thornthwaite and Hamon Drought Estimators Perform Similarly

Environmental analysis provided us with a set of non-redundant variables useful to describe long and short-term theoretical habitat drought stress. It was convenient to consider estimators based on potential evapotranspiration because of the conceptual power of this approach. Besides, it was appropriate to include the two main components from the subsets of bioclimatic variables related with precipitation or drought stress, because these emphasize temperature and precipitation, while other estimators only emphasize precipitation. These components allowed us to test their effect on the global analysis. Finally, it was practical to incorporate annual precipitation and precipitation of the driest period because these variables gave us a direct idea of long and short-term drought stress. All these estimators were congruent with visual inspections over precipitation maps for the area of geographical origin of the wild accessions. This is in line with the fact that bioclimatic variables are all different combinations of monthly air temperature and monthly total precipitation.

Some divergence between the Hamon and Thornthwaite models and tests with the different subsets of bioclimatic variables have demonstrated the possible ways to exploit environmental variability in order to infer different aspects of drought stress for the differed habitats. Therefore, the scope of the application will determine which metric is adequate. For example, the Thornthwaite drought index and the first component of the PCA analysis that used only bioclimatic variables directly associated with drought tolerance are good estimators of short term and sporadic drought stress. However, the Hamon drought index and the first component of the PCA analysis that used only precipitation variables are the best estimators of long-term drought stress. On the contrary, the second and third main components of the PCA analysis that included all the bioclimatic variables have low power and specificity to detect any kind of drought stress. Overall, robustness and resolution to discern between sub-populations were more extensive for the Thornthwaite and Hamon estimators than for the other two components. Thus, the former estimators should be preferred.

Some theoretical issues remain in order to guarantee the pertinence of each estimator. First, one must consider the link between habitat/geographical origin drought stress and plant drought tolerance. Two aspects modulate this relationship: 1) abiotic stress is a highly genotype×environment and plant species dependent phenomenon [12], [35], and 2) the collection site of a genotype in a semi-arid habitat does not make it necessarily drought tolerant. Several assumptions in the PET modeling must also be considered to access the boundaries in inferences made by the models [12]. Namely, our estimated drought stress is useful in a comparative perspective. It must not be used to make inter-specific comparisons because stress is a plant-specific perception and not a site characteristic, and because we are not including any soil water dynamics (by assuming that all precipitation water is potentially available to the plants). Another assumption is that plant distribution must be in equilibrium with niche requirements and ecological forces [36], [37], so that the errant presence of poorly adapted genotypes can be discarded.

A further consideration is the relationship between habitat ecology and drought stress. For example, precipitation patterns could be more related with the incidence of plant pathogens and the consequent biotic stresses than with drought stress [38]. To avoid this limitation we suggest rejecting estimators based on non-drought-related bioclimatic variables. Furthermore, we suggest using model-based estimators that consider the specific ways in which environmental variables can modulate drought stress. Hence, this is another argument to prefer Thornthwaite and Hamon drought indexes over the estimators derived from the PCA analysis.

In terms of selecting the best model, Thornthwaite and Hamon estimators were complementary. The Thornthwaite model takes into account latitudinal variation in addition of radiation and temperature [14], [16]. Meanwhile, the Hamon model focused on the latter two [15]. However, given the high correlation that is expected between day length, latitude, and seasonal temperature, the high consistency between both models does not turn surprising. In order to be able to consider both short and long term drought events, we also propose the use of the maximum monthly drought index and the normalized average drought index, keeping in mind the limitations described in the previous paragraphs.

In summary, we have estimated short and long term drought stress for the habitats and geographical origin of wild common bean accessions using multivariate methods and physiological (PET) modeling techniques. The habitat drought stress index based on the Thornthwaite and Hamon PET models are equivalent and are promising as predictors of overall drought tolerance. Recent examples illustrate how this resource should be coupled with considerations about population structure as a way to identify and exploit natural variation [39], [40], [41], [42], [43]. This will ultimately facilitate oncoming genealogical analysis and genome-wide genetic-environmental association studies that aim predicting fitness in wild populations [43], [44], [45].

## Supporting Information

Figure S1 **Geographic distribution for wild common bean accessions in relation with rainfall.** A. Wild common bean populations and precipitation in the driest period (mm) for the entire range of distribution, B. for Mexico, C. and for Peru, Bolivia and Argentina. D. Precipitation in the wettest period (mm) and total annual rainfall (mm).
(TIF)

Figure S2 **Dendogram of accessions constructed using the Pearson’s correlation coefficient and the middle joint method for all bioclimatic variables (P1–P19,** **table 1** **).** Accession names contain: accessions number+population assignation (Mesoamerican (Mexican wilds): K1, Guatemala: K2, Colombia: K3, Peru and Ecuador: K4, Andean (wilds from Argentina, Bolivia and southern Peru): K5), as defined by Blair *et al.* [26] and Broughton et al. [27]+quintiles for habitat drought stress (annual mean Thornthwaite Drought Index (DI), maximum Thornthwaite DI, annual mean Hamon DI, maximum Hamon DI). Branch colors are based on population structure. Red lines indicate groups of accessions with overall high quintiles.
(TIF)
