# Supplementary figures and images for: Drought Tolerance in Wild Plant Populations: The Case of Common Beans (Phaseolus vulgaris L.)
Source: PLoS One. 2013 May 3;8(5):e62898. doi: 10.1371/journal.pone.0062898 (PMC3643911; doi:10.1371/journal.pone.0062898)

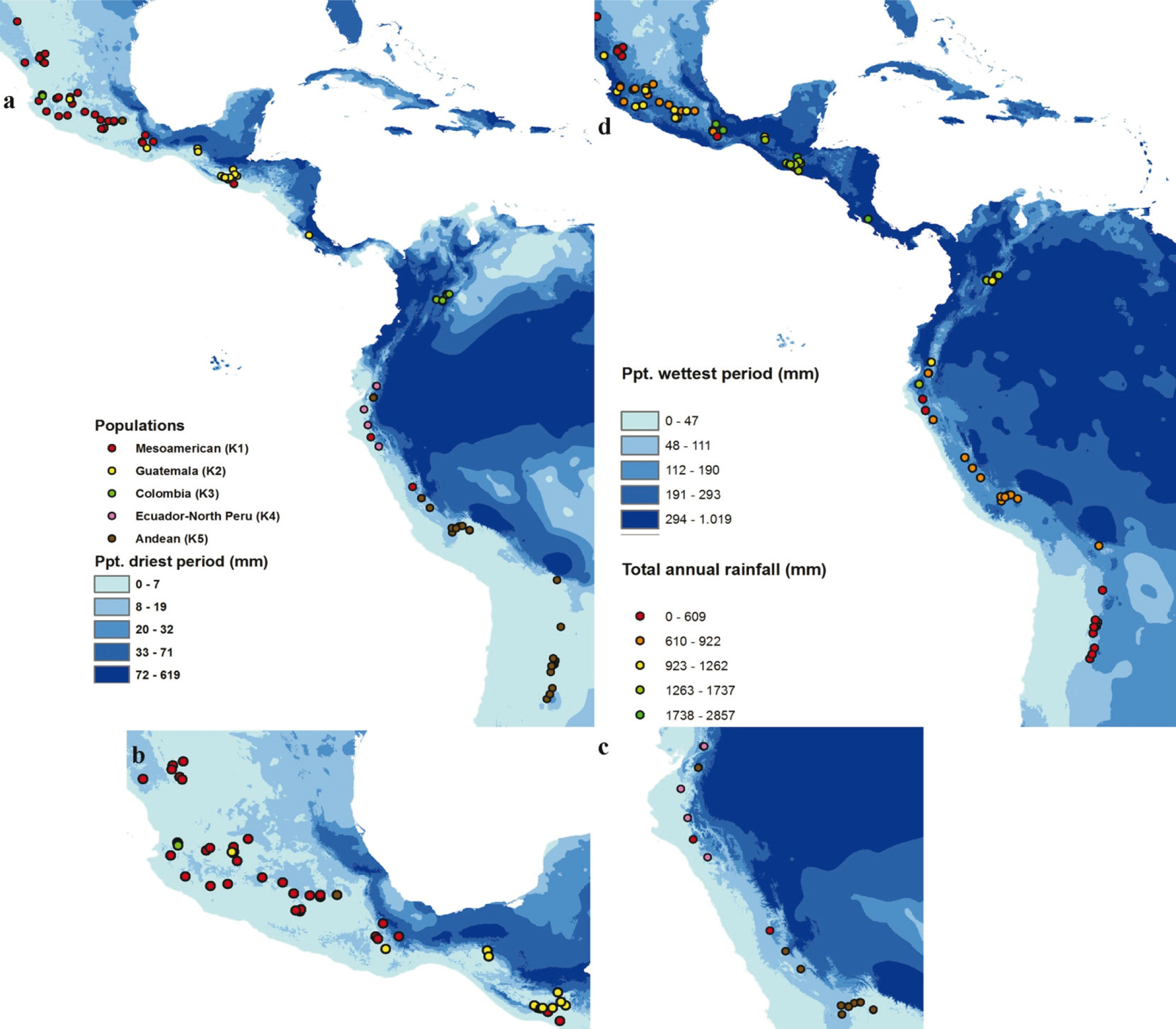

Supplement: Figure S1 — Geographic distribution for wild common bean accessions in relation with rainfall. A. Wild common bean populations and precipitation in the driest period (mm) for the entire range of distribution, B. for Mexico, C. and for Peru, Bolivia and Argentina. D. Precipitation in the wettest period (mm) and total annual rainfall (mm). (TIF) [file pone.0062898.s001.tif]

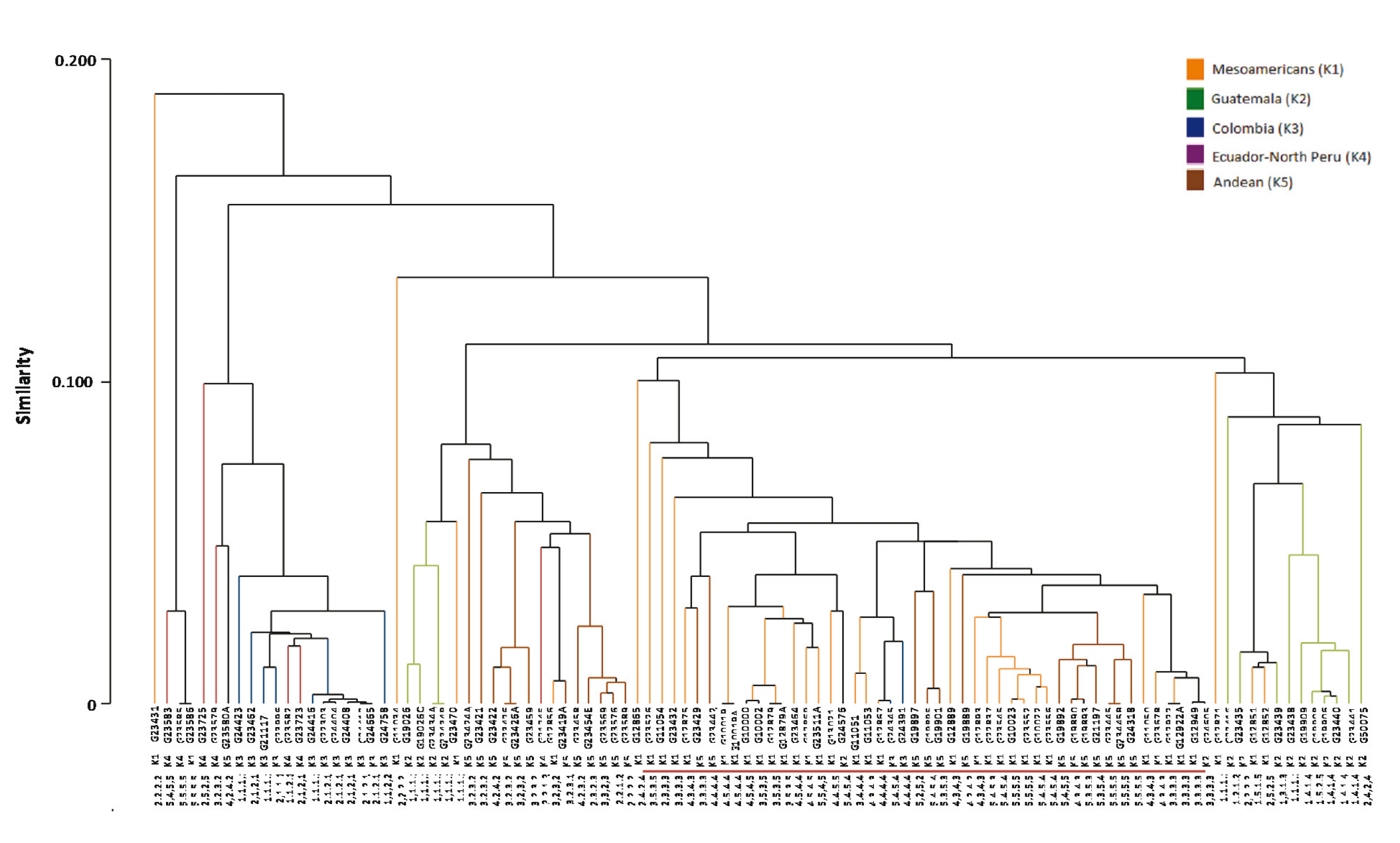

Supplement: Figure S2 — Dendogram of accessions constructed using the Pearson’s correlation coefficient and the middle joint method for all bioclimatic variables (P1–P19, table 1 ). Accession names contain: accessions number+population assignation (Mesoamerican (Mexican wilds): K1, Guatemala: K2, Colombia: K3, Peru and Ecuador: K4, Andean (wilds from Argentina, Bolivia and southern Peru): K5), as defined by Blair et al. [26] and Broughton et al. [27]+quintiles for habitat drought stress (annual mean Thornthwaite Drought Index (DI), maximum Thornthwaite DI, annual mean Hamon DI, maximum Hamon DI). Branch colors are based on population structure. Red lines indicate groups of accessions with overall high quintiles. (TIF) [file pone.0062898.s002.tif]
